# Lymphatics in health and disease: a new thematic series in vascular cell

**DOI:** 10.1186/2045-824X-5-14

**Published:** 2013-07-25

**Authors:** Jan Kitajewski, Carrie J Shawber, Michael Simons

**Affiliations:** 1 Pathology, Columbia University Medical Center, NY, NY, 10032, USA; 2 OB/GYN, Columbia University Medical Center, NY, NY 10032, USA; 3 Herbert Irving Comprehensive Cancer Center, Columbia University Medical Center, 1130 St. Nicholas Ave, NY, NY 10032, USA; 4 Yale Cardiovascular Research Center, Yale University, New Haven, CT 06511, USA

## Abstract

Vascular Cell is launching new series on lymphatics, a vascular system required for physiological fluid balance and immunity, and whose damage leads to edema.

## Editorial

It has been over a century since the mammalian lymphatic system was first described in precise anatomical graphical presentations [1]. Since that time, we have seen dramatic advances in our description of lymphatics. We can now read numerous published reports that provide fascinating details of the molecular, cellular, and functional aspects of lymphatics. Lymphatic researchers have achieved a molecular precision by which they can now describe lymphatic development, function, and lymphatic diseases. Vascular Cell announces the launch of a thematic series on “Lymphatics in health and disease”. This series comes at a time when the field has matured and is ripe for translation into treatments for lymphatic disorders. This thematic series will provide reviews from experts on the study of the biology of lymphatic vasculature and on the role of lymphatic vessels in disease states such as edema, inflammatory diseases, and cancer.

The lymphatic vascular system functions to return tissue fluid, macromolecules, and cells back to the blood circulation. During mammalian embryogenesis, formation of the lymphatic vascular system is an early and essential process. Lymphatics develop by sprouting from the lymph sacs, which originate from the embryonic veins, thus lymphatic development follows arterial-venous specification. Lymphangiogenesis proceeds in parallel with angiogenesis, generating tissues rich in both blood and lymphatic vessels.

Disruption of the lymphatic system can occur due to inherited mutations in affected individuals or secondarily as a result of surgery. When lymphatics do not form or function properly, drainage of interstitial fluids is reduced and fluid accumulates, causing what is referred to as lymphedema. The causes of lymphedema can include an insufficient number of lymphatic vessels, reduced lymphangiogenesis, impaired function of lymphatic valves, loss of vascular smooth muscle cells, or obstruction of lymphatic vessels. Reduced lymphatic function ultimately leads to accumulation of the protein-rich lymph fluid with contains proteins, imflammatory cells and lipids. The genetic basis of some congenital hereditary lymphedemas is known and is often associated with mutations in gene region encoding the tyrosine kinase domain of VEGFR-3. During tumor growth, lymphatics can be recruited to extra- or intra-tumoral sites and contribute to the pathology by serving as a conduit for metastatic cells.

A variety of topics will be covered in this thematic series, a list of which could derive from session topics of a recent meeting on lymphatics sponsored by Yale University and the North American Vascular Biology Organization or NAVBO (see http://www.navbo.org/lymphatics) or an upcoming 2014 Gordon Conference entitled Molecular Mechanisms in Lymphatic Function & Disease: State-of-the-Art in Lymphatic Research and Biology. We invite reviews that cover the growth factors, receptors, effectors and transcriptional regulators that drive the specification and formation of the lymphatics during development and in adult tissues [2]. Descriptions are needed on how lymphatics function to promote fluid and lipid absorption, maintain directional flow and participate in immune surveillance. A discussion of lymphatic dysfunction due to genetic deficiencies or surgery-induced damage should be considered in order to better chart the path toward repair [3]. One must also consider that tumors can recruit lymphatics, tumor cells can enter the lymphatic system and spread to lymph nodes [4]; events critical to both diagnosis and possibly treatment of metastatic spread. Finally, other functional aspects of lymphatic circulation such as an emergent area of blood pressure control [5] can be considered.

To submit your manuscript, please use the online submission system for Vascular Cell and indicate in your cover letter that you would like it to be considered for the thematic series on lymphatics. Alternately, please send a pre-submission enquiry to either Jan Kitajewski at jkk9@columbia.edu, Carrie Shawber at cjs2002@columbia.edu, or Michael Simons at michael.simons@yale.edu. Go with the flow and contribute to this thematic series on the amazing lymphatic vascular system.
